# “None of us are lying”: an interpretive description of the search for legitimacy and the journey to access quality health services by individuals living with Long COVID

**DOI:** 10.1186/s12913-023-10288-y

**Published:** 2023-12-12

**Authors:** Katelyn Brehon, Maxi Miciak, Pam Hung, Shu-Ping Chen, Kadija Perreault, Anne Hudon, Marguerite Wieler, Simone Hunter, Lance Hoddinott, Mark Hall, Katie Churchill, Darren A. Brown, Cary A. Brown, Geoffrey Bostick, Kate Skolnik, Grace Lam, Jason Weatherald, Douglas P. Gross

**Affiliations:** 1 University of Alberta, Edmonton, Canada; 2 Université Laval, Quebec City, Canada; 3 University of Montreal, Montreal, Canada; 4 BreatheWell Physiotherapy, Calgary, Canada; 5 Alberta Health Services, Calgary, Canada; 6 University of Toronto, Toronto, Canada; 7 Chelsea and Westminster Hospital NHS Foundation Trust, London, England, UK; 8 University of Calgary, Calgary, Canada

**Keywords:** Lived experience, Healthcare access, Canada, Access barriers, Quality, COVID-19

## Abstract

**Background:**

Understanding of Long COVID has advanced through patient-led initiatives. However, research about barriers to accessing Long COVID services is limited. This study aimed to better understand the need for, access to, and quality of, Long COVID services. We explored health needs and experiences of services, including ability of services to address needs.

**Methods:**

Our study was informed by the Levesque et al.’s (2013) “conceptual framework of access to health care.” We used Interpretive Description, a qualitative approach partly aimed at informing clinical decisions. We recruited participants across five settings. Participants engaged in one-time, semi-structured, virtual interviews. Interviews were transcribed verbatim. We used reflexive thematic analysis. Best practice to ensure methodological rigour was employed.

**Results:**

Three key themes were generated from 56 interviews. The first theme illustrated the rollercoaster-like nature of participants’ Long COVID symptoms and the resulting impact on function and health. The second theme highlighted participants’ attempts to access Long COVID services. Guidance received from healthcare professionals and self-advocacy impacted initial access. When navigating Long COVID services within the broader system, participants encountered barriers to access around stigma; appointment logistics; testing and ‘normal’ results; and financial precarity and affordability of services. The third theme illuminated common factors participants liked and disliked about Long COVID services. We framed each sub-theme as the key lesson (stemming from all likes and dislikes) that, if acted upon, the health system can use to improve the quality of Long COVID services. This provides tangible ways to improve the system based directly on what we heard from participants.

**Conclusion:**

With Long COVID services continuously evolving, our findings can inform decision makers within the health system to better understand the lived experiences of Long COVID and tailor services and policies appropriately.

**Supplementary Information:**

The online version contains supplementary material available at 10.1186/s12913-023-10288-y.

## Background

Emerging evidence suggests that globally, 30–50% (dependent on ethnicity, sex, and hospitalization status) of those who contracted Coronavirus Disease 2019 (COVID-19) experience Long COVID symptoms [1], with an estimated 144.7 million people living with Long COVID globally [2]. Long COVID was a term originally created by patients as there was no term to describe the symptoms they were experiencing post-acute COVID-19 infection [3]. The World Health Organization defines Long COVID as a condition: “occur[ing] in individuals with a history of probable or confirmed SARS CoV-2 infection, usually 3 months from the onset of COVID-19 with symptoms that last for at least 2 months and cannot be explained by an alternative diagnosis” [4]. However, there is no consensus on one definition [5].

Long COVID is more commonly reported by women [1, 6–9], older individuals [1, 10, 11], individuals with pre-existing comorbidities [12, 13], and those who experienced more severe acute illness [1, 10, 14]. A diverse spectrum of health challenges have been described including physical (e.g., fatigue or exhaustion), cognitive (e.g., memory challenges), and mental health symptoms (e.g., post-traumatic stress), which have notably impacted social participation and overall functioning [7, 15–20]. Understanding of Long COVID has advanced in part due to patient-led initiatives; discussions about prolonged symptoms after COVID-19 first emerged through patient discussions on social media. An all-patient team published the first survey of prolonged symptoms [21] and a patient-led research team completed the first cohort study of Long COVID [19]. In November 2020, a group of physiotherapists from the United Kingdom and the United States founded Long COVID Physio: an international, patient-led partnership collaborating to advocate for those living with Long COVID and working to advance policy [22]. Long COVID Physio has played a critical role in developing research-informed guidance for Long COVID rehabilitation [22]. In June 2021, Long COVID Physio partnered with World Physiotherapy to develop a briefing paper on safe rehabilitation approaches for individuals living with Long COVID [23].

In other contexts, barriers to health services included geographical inaccessibility and lack of funding [24, 25]. In patients with other complex chronic illnesses, such as myalgic encephalomyelitis/chronic fatigue syndrome, geographic and financial barriers are present as well as barriers in access to specialist care [26]. Previous qualitative scholarship has identified lack of knowledge and guidance from providers, patients not being believed by the healthcare system, lack of health literacy, fragmented services, general busyness of the system, distrust in providers, and language barriers as barriers to access for individuals with Long COVID [27, 28]. Pandemic-related travel and gathering restrictions likely exacerbated barriers for at-risk populations including rural, Indigenous, and low-income groups [24, 25]. In Alberta, a province with a single, publicly-funded health authority, many community rehabilitation staff were redeployed to support the COVID-19 response in other areas (i.e., testing, contact tracing, inpatient care). To help reduce the impact of Long COVID symptoms on functioning, disability, and health, Long COVID services (including rehabilitation) have been established in Alberta, Canada within public [29–32], private [33], and Workers’ Compensation (i.e., a provincial, government legislated insurance program providing wage replacement and healthcare services for employees experiencing work-related health conditions) systems [34, 35]. However, to our knowledge, no research analyzing the need for, access to, and quality of, such services exists in an Albertan context.

Levesque et al.*’s* “conceptual framework of access to health care” informed our study design [36]. This framework views access to health services as a dynamic interaction between patients’ abilities to perceive, seek, reach, pay, and engage in healthcare services and aspects of the health system (approachability, acceptability, availability and accommodation, affordability, and appropriateness) with both influencing access to, and utilization of, healthcare [36]. We used the framework to 1) initially conceptualize how we considered access and health service engagement given the complexity of the phenomena and 2) at an operational level to inform the interview guide (more details included below). This framework has been previously used in health services [37, 38], rehabilitation [39, 40], and global health [41] research. We used this framework because it provided a comprehensive overview of the complex phenomenon of access to patient-centred care within systems, considering the breadth of both ecological levels as well as individuals’ diverse experiences of accessing care [36]. Although comprehensive, the framework was not prescriptive. These characteristics were coherent with our pragmatic methodological approach.

### Study objectives

Building on Levesque et al.*’s* (2013) “conceptual framework of access to health care” [36], we aimed to better understand the need for, access to, and quality of, health services for people living with Long COVID in Alberta. We define Long COVID health services as any diagnostic, medical, and/or rehabilitation service(s) participants accessed (or attempted to access) to try to improve their Long COVID symptoms.

## Methods

Ethics approval was obtained from the University of Alberta’s Health Research Ethics Board. All participants provided informed consent. No ethical challenges were encountered while conducting the study.

### Study design

We used Interpretive Description (ID) [42], a qualitative methodological approach partly aimed at informing clinical decisions. ID merges researchers’ disciplinary knowledge in guiding the project and patient lived experiences to provide clinically useful findings. The flexibility of the approach and its alignment with the constructivist paradigm enables deep exploration of questions addressing complex, practical problems [43].

### Study population

We sought Albertans with Long COVID who accessed services through Alberta’s Rehabilitation Advice Line (RAL) [32], public physician-led Long COVID clinics [29–31], a specialized, private physiotherapy clinic (BreatheWell Physiotherapy) [33], and a multidisciplinary clinic contracted to provide services to Workers’ Compensation patients experiencing Long COVID (Millard Health Post-COVID Clinic) [34]. We also included a group of people with Long COVID who had accessed little to no services to learn from those who may have experienced barriers significant enough to prevent them from accessing care. These individuals contacted the researchers after reading an online news article. We recruited from these groups to provide a broad array of perspectives from individuals who live rurally or in urban centres as well as those who had accessed public, private, or little to no services.

#### Inclusion/exclusion criteria

Inclusion criteria were: people reporting ongoing symptoms at least three months after confirmed or presumed COVID-19 infection; age ≥ 18 years; able to participate in English; and able to provide informed consent. Participants did not need to have a positive polymerase chain reaction (PCR) test. Those reporting only long-term alterations in taste and/or smell were excluded as services to address these symptoms are not widely available and are mainly self-directed [44].

### Recruitment

Purposive sampling towards maximum variation directed recruitment. The three primary diversification criteria were age, gender, and geographical location (rural/urban). We aimed for 50 to 60 participants distributed across the five Long COVID recruitment settings. This sample size was considered sufficient for qualitative analysis in an ID study with a diverse population [45, 46]. Recruitment at each site was led by staff liaisons who approached participants who met study inclusion criteria to introduce the study and gain participants’ consent to share their contact information with the research team. The study coordinator (KB) then contacted each interested participant to tell them more about the study, gain their informed consent, and schedule an interview.

There was an enthusiastic response to recruitment with the research team having to turn some interested participants away for study feasibility purposes. The high level of interest in this study may have been due to the novelty of Long COVID and that individuals living with Long COVID wanted to support research to increase understanding of the condition.

### Data collection

Interviews were completed by one experienced interviewer (KB) and one PhD-level trainee (PH). We conducted remote, 1:1, semi-structured interviews by videoconference (Zoom) or phone. The interview process was flexible (i.e., could be divided into multiple sessions, if necessary) to accommodate symptoms experienced by individuals living with Long COVID, such as fatigue or difficulty with sustained attention. We divided two interviews into multiple sessions to accommodate participants.

We used a semi-structured interview guide informed by patient partners and the Levesque et al. (2013) framework [36]. The framework was used to help us conceptualize various aspects related to access, such as affordability, that should be included in the interview guide. However, the interview guide also included questions deemed important by patient partners and other members of the research team. Specific interview topics addressed health concerns, perception of service needs, access to services, and service quality. Probing questions were used to elicit greater description, as necessary.

At the end of interviews, participants were asked open-ended questions about how they would describe their gender, cultural or ethnic background, and place of residence (urban/rural). Vaccination status at time of infection was determined either through disclosure by participants or date of infection (most participants were from waves one and two and vaccines were not widely available until the third wave in Alberta). All interviews were recorded, transcribed verbatim, and reviewed for accuracy.

### Data analysis

Our analysis was informed by Braun and Clarke’s six phases of reflexive thematic analysis [47, 48]. Transcripts were imported into NVivo data management software [49]. A subset of researchers versed in qualitative methods with backgrounds in clinical rehabilitation, public health, and health services (hereby referred to as the analysis team) met three times throughout the analysis. The first meeting was used to plan the analysis. The primary study analyst (KB) reviewed the transcripts for accuracy and read through all interview transcripts conducted by PH. Initial codes were generated by KB and PH (depending on who conducted the interview) inductively for 20 interviews and high-level interview synopses (i.e., 2–3-page overviews) were written and shared with the analysis team. The analysis team reviewed the synopses and met a second time to collate codes and ideas into initial themes.

The remaining transcripts were coded by KB while remaining receptive to novel codes. The codes and initial themes were then considered in relation to one another and collapsed or expanded based on patterns of meaning. Interview synopses for the remaining 36 transcripts were written and shared with the analysis team who met one final time to review themes and sub-themes to ensure they were congruent with the insights gleaned from synopses. Themes and sub-themes were then named and defined. Analysis continued while the final report was being drafted and included feedback from patient partners on themes, key quotes, and presentation of ideas. KB and PH met frequently throughout the analysis to review, refine, and confirm codes and themes of selected interviews, ensuring credibility of interpretations.

Braun and Clarke highlight the importance of researchers using reflexive thematic analysis as a starting point with flexibility to make it their own [47, 50]. However, they note it is important to outline exactly how methods diverge from their account of reflexive thematic analysis with justification for doing so [47, 50]. As such, we note that while our analysis was informed by Braun and Clarke [47, 48], we made two adaptations. We used an analysis team, leveraging our research team's diverse disciplinary backgrounds to ensure findings were relevant to practice and policy decision-making. Given the number of interviews and scope of the data, we also shared interview synopses with the analysis team to make review more comprehensive and feasible.

### Rigour

Rigour was promoted through an audit trail of decisions for accountability; open-ended questions to prioritize participant voice; thick description; collaborative coding for discussion of subjectivity and openness in analysis; as well as reflexive journaling and discussion [51].

## Results

### Participant characteristics

We completed 56 interviews. Table 1 outlines participant characteristics, with additional details about participants’ ethnicity in Additional file 1: Appendix A. Participants primarily identified as female (60.7%). Our sample ranged from 20 to 74 years of age with a mean (standard deviation) age of 49.29 years (13.04). Twenty-one (37.5%) participants had pre-existing conditions including asthma, multiple sclerosis, and diabetes, for example. The majority (76.8%) were from urban or suburban centers. Participants had diverse education and occupational backgrounds. Some worked in front-line positions (public facing jobs deemed essential workers for the community) during the pandemic (e.g., healthcare, education, retail, etc.). Others worked either in non-front-line positions (e.g., jobs without interaction with the public such as management, home maker, etc.), were unemployed, retired, or chose not to disclose their employment status. We did not attempt to characterize individuals’ severity or length of Long COVID symptoms since symptom severity varies considerably from person to person and testing was variably accessible for participants (i.e., some did not have a positive test to indicate when they contracted the virus).

**Table 1:** Study sample demographic characteristics by recruitment site and in total

	Recruitment Site					
	**Public Long COVID Clinics**	**BreatheWell Physiotherapy**	**Rehabilitation Advice Line**	**Workers’ Compensation Clinic**	**News Article Recruitment**	**TOTAL**
Gender						
Male	5	1	4	5	7	22
Female	9	8	4	9	4	34
Mean Age	50.6 years (min 20; max 74)	51.0 years (min 34; max 69)	52.1 years (min 35; max 74)	51.8 years (min 37; max 63)	40.0 years (min 24; max 67)	49.3 years
Mean Interview Length	42.2 min	61.0 min	57.1 min	56.8 min	46.1 min	52.6 min
Rural/Urban						
Rural	6	2	2	1	2	13
Urban/ Suburban	8	7	6	13	9	43
Ethnicity						
White	9	8	7	9	9	42
Ethnic Minority	5	1	1	5	2	14
Vaccination Status at Time of Infection						
Unvaccinated	13	9	7	11	10	50
One Dose	0	0	0	3	1	4
Undisclosed	1	0	1	0	0	2
Alberta COVID-19 Wave Where Participant Was Infected						
Wave 1 (March 2020 to June 2020)	0	7	3	1	1	12
Wave 2 (July 2020 to February 2021)	11	2	3	11	6	33
Wave 3 (March 2021 to June 2021)	3	0	2	2	3	10
Wave 4 (July 2021 to December 2021)	0	0	0	0	1	1

### Themes

**Fig. 1:**
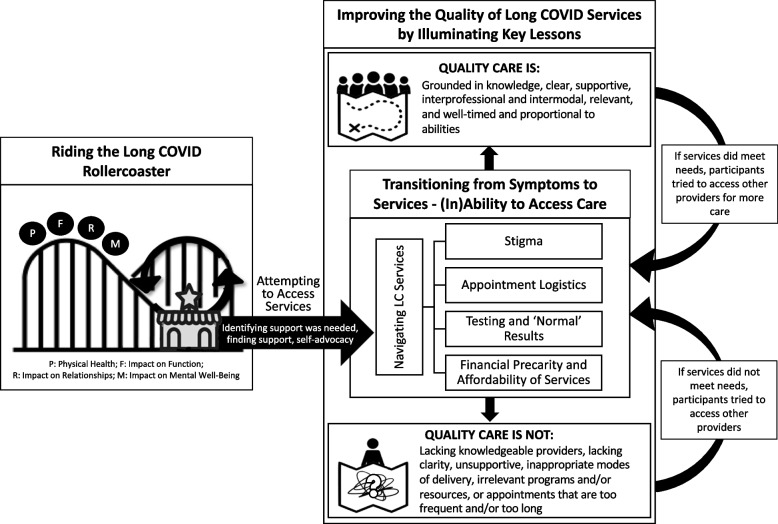
Thematic figure depicting relationships between themes and sub-themes

Three key themes were generated: (a) Riding the Long COVID Rollercoaster; (b) Transitioning from Symptoms to Services – (In)Ability to Access Care; and (c) Improving the Quality of Long COVID Services by Illuminating Key Lessons. The relationships between these themes and corresponding sub-themes are depicted in Fig. 1. Full quotes are included in Additional file 2: Appendix B.

#### Theme A: Riding the Long COVID rollercoaster

In theme (a), participants spoke about the relapsing–remitting, “rollercoaster”-like nature of their Long COVID symptoms and the resulting impact on functioning, disability, and health. As expected, the ongoing symptoms participants’ experienced were highly diverse: “it affects everybody […] to different extents and everybody has different symptoms, which is really hard because it's just a myriad of symptoms” (female participant, private clinic). However, some common symptoms were mentioned including: fatigue or exhaustion, dyspnea, cognitive impairment, tachycardia, chest pain, body and joint pain, and headaches.

The uncertain and unstable trajectory of symptoms affected each participant’s level of functioning and health differently. Participants were left feeling like “a shell of the person [they] used to be” (female participant, public clinic) and as if they were experiencing “paranoia over not knowing [their] own body” (male participant, recruited through news article).

*Impact on function*

**Activities of Daily Living (ADLs)**

Prior to COVID-19, most participants were not experiencing any functional challenges. With Long COVID, participants reported that attempting ADLs would oftentimes exacerbate symptoms and were avoided, if possible. Basic ADLs (e.g., mobility and person hygiene activities) and instrumental ADLs (e.g., cleaning, home maintenance, and cooking and meal preparation) requiring more physical and cognitive exertion were identified as most challenging. Many participants used strategies to manage their energy when completing ADLs such as pacing, energy or activity management, taking frequent breaks, or only focusing on one activity a day, while some needed to use newly acquired assistive devices (i.e., stool, walker, cane) to function independently. Some participants required assistance from friends or family or accessed paid services to help manage ADLs, particularly related to household activities, cooking, and shopping. This left them feeling “like a burden” because they could not “do a lot around the house” (male participant, Workers’ Compensation).

“I've had to really prioritize what's the most important things to do and how I need to space that through the week. And … I have to really break down my tasks and to small steps like if I go down and start laundry, I can't make a bunch of trips going down to fill the washer because I get really short of breath and tired. If I grab laundry out of the dryer and carry it up the stairs, by the time I get upstairs I am so short of breath and exhausted, I put on my bed, and I go and I go sit my chair and I rest for about a couple hours. So … I've had to figure out ways to break up the tasks … Because … it's tiring” (female participant, Workers’ Compensation)

**Work**

The “rollercoaster” of symptoms had a notable impact on participants’ ability to work. Some participants were not working prior to infection due to living with a pre-existing disability or being retired. For those who were able to return-to-work, some had supportive employers who offered gradual and flexible return-to-work plans and/or modified duties. In these instances, the return-to-work process was reported as positive and most often successful. Those without an accommodating employer or who could not perform their previous job duties often had to look for alternative work environments or stop working altogether. Almost half of participants found themselves unable to work due to ongoing symptoms which snowballed into feelings of uncertainty or worry about the future.

“I’m still nowhere from being able to return-to-work which is really hard to deal with. I loved my job and I really … hoped that I could return to it, but I’m not sure … that is going to be a reality for me … maybe I’ll be able to return to something … but, I need something that's going to be really flexible” (female participant, private clinic)

**Sleep**

Although some participants thought they were sleeping more due to fatigue or had no changes to their sleep, most participants reported sleep disturbances. Some experienced trouble falling asleep, woke frequently, and/or had breathing difficulties. To cope with sleeping difficulties, some participants tried over-the-counter sleeping medications or supplements. The most common coping strategy was napping during the day to manage the severe exhaustion caused by Long COVID; even those who slept well during the night often had to nap during the day.

“… it takes me two sleeping pills, three sleeping pills … to be able to go to sleep and I don't stay asleep and it's not really a deep sleep ever … I … g[e]t maybe an hour and a half of REM a night … so I just wake up exhausted … every single day.” (female participant, public clinic)

##### Impact on relationships

Many participants discussed the importance of having a social support system during their Long COVID journeys. Support systems included spouses, friends, parents, and children who assisted participants with managing ADLs, supported them financially if they were unable to work, and acted as emotional support. Some participants lacked a support network making their experience “actively isolating” (male participant, recruited through news article). There were also feelings of guilt for not being able to fulfill their social roles: “it impacts … every single facet of your relationships, your ability to maintain connections outside of the home with friends, your ability to … be a father, ability to be a supportive partner and husband” (male participant, recruited through news article). These feelings provoked negative family interactions, trying to push through symptoms, and adverse effects on mental well-being.

##### Impact on mental well-being

The effects of Long COVID on mental well-being were severe and far-reaching; most participants reported feeling “angry”, “anxious”, “depressed”, “frustrated”, “stressed”, “desperate”, and/or “helpless”. We want to stress that psychological diagnostic labels such as “anxiety” or “depression” should not be flippantly used to characterize the experiences of individuals living with Long COVID. Long COVID is not analogous to these terms and treating it as if it is leads individuals to feel as though their experiences are being minimized.

Participants struggled to adjust to the disabling and uncertain nature of Long COVID. Many participants wrestled with having a poorly understood “invisible illness”. While deemed essential for learning to cope with and manage mental well-being, only a subset of participants were offered 1:1 psychological counselling as part of their Long COVID program (e.g., through Workers’ Compensation):

“when … your life is not normal anymore and when you have … an invisible disease … it's hard because people … can't see that you're ill … So the psychologist … has been … critical because I am not depressed, but I can see how this would push a person into a depression. I'm sad, … I'm angry, I'm frustrated … but I'm not depressed, but I have seen people [online who have] lost their jobs, …all their money, … their homes. So psych[ological] support to help people [is] critical.” (female participant, Workers’ Compensation)

All other participants were either not offered psychological support, already saw a therapist on a regular basis, sought counselling on their own, were discouraged from seeking publicly-funded psychological help due to wait times, did not want counselling on their health record, or did not feel it was necessary.

#### Theme B: Transitioning from symptoms to services – (in)ability to access care

*Attempting to access services*

Initial access to services was dependent on what symptoms and functional changes drove participants to seek care, the guidance and advice received from a medical professional or trusted individual, and/or self-advocacy efforts.

**Identifying that support was needed**

Most participants explicitly spoke about reasons prompting them to look for Long COVID services. The most frequently mentioned were addressing invisible cognitive symptoms, improving breathing, return-to-work, return to pre-COVID levels of function, and learning to manage fatigue. Other reasons for seeking care included improving strength and mobility and trying to find definitive answers about Long COVID. Participants needed to be health literate to understand that there may be services available that could help them address their concerns as well as where and how to access services: “Even knowing about it. That's the barrier … I had no clue … I never even thought these things existed. I just thought I was … going to have to do it myself.” (female participant, recruited through news article).

**In(Ability) to find support**

Many did not know what Long COVID services were available in the province, how to navigate to them, or did not think there would be any support available yet. The majority of individuals sought guidance from a medical professional (nearly all sought guidance from their family doctor) or someone with greater experience in the health system. For some, these interactions facilitated access to services. Having an accepted Workers’ Compensation claim and being assigned a personal case manager also facilitated access. However, if family doctors did not suggest additional services, participants often went no further within the system:

“It's … not knowing who to call. … my family doctor is fish out of water like there is no 1-800-post-COVID helpline … right? … [T]here's a lot of people out there and they're bouncing around … trying to manage on their own … just don’t know who to go to anymore” (male participant, private clinic)

**Self-advocacy**

Family doctors’ limited experience with Long COVID led many participants to feel belittled and/or not believed following interactions. Some reported their symptoms were attributed to anxiety, depression, or panic attacks. As a result, many participants felt they needed to self-advocate for acceptance, recognition, and access to better care. Self-advocacy manifested itself in participants asking for specific referrals, self-referring, trying various self-management strategies, or seeking advice from peers living with Long COVID. The success of self-advocacy efforts varied between participants. Some were able to “build [their] own care team” (female participant, private clinic) and felt like they were making progress in recovery. Others felt as though they had to “fight tooth and nail” (male participant, Workers’ Compensation) for care received. Given that a hallmark symptom of Long COVID is fatigue or exhaustion, many participants wished they did not need to expend limited energy on self-advocacy:

“I think finding the right doctors and the right people to help with … the hard part [is essential] and when was as sick as I am and was, … I just wish I didn't have to do that myself.” (female participant, Workers’ Compensation)

*Navigating Long COVID services*

Barriers related to stigma; appointment logistics; testing and ‘normal results’; and financial precarity and affordability of services existed when trying to navigate Long COVID services within the health system.

**Stigma**

The perception that those living with Long COVID were still contagious was frequently mentioned as a factor decreasing participants’ comfort accessing services. Many participants were met with hesitancy when going for testing or appointments even though they were past the contagious period of the virus, leaving them feeling embarrassed:

“I was going for my follow-up … lung test … And [the tech] … looked at the requisition and … said you have COVID. … [And] in front of everybody [she said] … you have to go home … you have COVID and I was mortified … it's scary enough to have COVID. [T]hen to be treated like you have the plague after … you're not contagious anymore, … it was really very bad.” (female participant, public clinic)

Multiple participants experienced weight stigma: their providers attributed ongoing symptoms to their weight or “fat-shamed” them (female participant, private clinic). Age-related stigma was discussed by some younger participants who felt as though their symptoms were not taken seriously presumably because younger people were not supposed to experience severe symptoms. Self-stigma of having contracted COVID-19 was also present. All of these forms of stigma created feelings of shame and made some participants hesitant to access Long COVID services.

**Appointment logistics**

Appointment wait times, driving and transportation, and symptoms resulting in being physically unable to leave the house were often mentioned as logistical barriers to access. While waiting for health appointments was common prior to the pandemic, staff redeployment, timing of Long COVID program development, and referral uncertainties and errors increased time delays. Driving and transportation posed a barrier to access as many participants were not comfortable driving to and from appointments due to their symptoms. Having the energy to get up, get ready, and leave the house was challenging for many, therefore attending appointments outside the home was viewed as unfeasible. Some participants appreciated having virtual or in-home care options as they did not have to use their limited energy to get to appointments:

“Some … things were virtual so that really helped me [so I didn’t] have to drag [my] butt out there because getting up in the morning is really hard. And then as you go through the week, you're just more and more tired as you go to the things” (female participant, Workers’ Compensation)

**Testing and ‘normal’ results**

Participants conveyed concerns regarding how testing (i.e., PCR) and the subsequent results influenced their ability to access the right services at the right time. While presumed COVID-19 cases are now included in clinic eligibility criteria in Alberta and recognized globally [4], many participants wondered whether the lack of positive PCR testing and minimal care early in their journey contributed to ongoing symptoms. Participants who lacked a positive PCR test often felt “desperate to prove [they] had something” (female participant, private clinic) and pushed themselves during health assessments (i.e., treadmill stress tests) sometimes causing post-exertional symptom exacerbation (also known as post-exertional malaise) [52–54]. Participants also reported frustration with perceived futile diagnostic testing, saying the test results did not match their symptoms and that receiving ‘normal’ test results created further limitations to accessing other providers:

“I had [all] those [tests] done and, like so many others, … these tests are showing basically nothing. And it’s like, ‘oh, your heart, … looks … fine.’ But I’m here to tell you it’s not. None of us are lying. … the tests … that you're using are ineffective … It doesn't mean that we don't have this, it just means that you don't have the means to detect it.” (male participant, recruited through news article)

**Financial precarity and affordability of services**

Many participants discussed how having a lower income due to being unable to work prevented them from accessing services since less money was coming in while their household expenses had increased. Beyond Long COVID services, these expenses included grocery or meal delivery, parking at appointments, equipment and assistive devices, and supplements or medication. For some, being unable to work was not stressful as they had financial resources. Others had income support through Workers’ Compensation, Employment Insurance, or long- or short-term disability insurance. Many participants did not qualify for government-provided COVID-19 income support which increased financial precarity and the inability to afford Long COVID services. Financial precarity was further exacerbated if participants were the primary provider and if they were unsure whether they would be able to return to their job.

“I’m … still making [Workers’ Compensation] money which is less than what I was making before to the point where … our budget, we only have like $300 … to spare and most of that is go[ing] to covering overdue and late charges on bills that we've put off because we didn't have the money to pay them … Like we're stretched extremely thin” (male participant, Workers’ Compensation)

#### Theme C: Improving the quality of Long COVID services by Illuminating key lessons

The barriers experienced when attempting to transition from symptoms to services discussed above influenced which service(s) participants accessed. Participants discussed factors about Long COVID services that they liked (i.e., enhancing service quality) and/or disliked (i.e., diminishing service quality). We framed each sub-theme as the key lesson related to quality that stemmed from all related likes and dislikes. Framing each sub-theme as an actionable opportunity ensures the findings are interpreted with the overarching goal of providing tangible ways to improve the system based directly on what was heard from participants.

*Quality care is grounded in knowledge*

Participants often spoke about how providers had limited knowledge about Long COVID treatment and rehabilitation. Many accepted this limited knowledge since COVID-19 is a novel illness whereas others disliked this limited knowledge and expected providers to have more definitive answers:

“nobody really knows right? Everybody was still just trying … and [saying] we'll try this and we'll do that, we'll do this, so I think, for me because … I'm so scientific and … like to have black and white answers, I’m like if I do this, I want this outcome right, so I think that was hard” (female participant, Workers’ Compensation)

Regardless of whether participants were accepting or intolerant of this limited knowledge, most believed there was an opportunity to provide more education and/or support for providers to increase knowledge around Long COVID management. Participants noted family doctors specifically needed additional education and support to recognize symptoms of Long COVID, make appropriate referrals, and direct participants to available self-management resources.

##### Quality care is clear

Clarity regarding program components, available resources and information, and next steps in care plans translated into perceived quality of Long COVID services. Participants wanted to know what to expect from the program(s) they accessed. Some participants reported being misinformed about what symptoms the programs addressed leading them to access programs they did not need. Participants felt that self-management education and resources needed to be provided in manageable amounts, understandable, and easy to access to be perceived as clear. Cognitive issues related to Long COVID amplified this need. If participants felt overwhelmed by the amount of information or if it was difficult to access, they often did not use it:

“she sent me so much stuff by email I didn’t know if I was coming or going. Like there was pages and pages and pages of stuff and some of it was out of my realm of understanding, … I didn't read a lot of that stuff … because I found [it] … hard to wade … through” (male participant, RAL)

Written self-management resources do not serve as a substitute for dialogue and need to be supplemented with follow-up discussions where providers check patient understanding and provide verbal explanations. Finally, participants perceived care as quality when they had clear next steps in their care plan. Clarity in next steps was improved through regular follow-ups and check-ins with providers.

##### Quality care is supportive

Participants felt supported by their providers if they were knowledgeable, validated their experiences and concerns, made them feel understood, and reassured them. Participants wanted their providers to help them feel as though they were “not in it alone”:

“the message that I would like to communicate to all health care providers out there is to listen to your patients and the symptoms that they're experiencing, and don't disregard them, and don't tell them that it's just in their heads. The anxiety … that lots of people are feeling isn't what caused their illness. It's their illness and people not believing them that causes more anxiety.” (female participant, private clinic)

Participants disliked feeling belittled, dismissed, and/or not heard. Participants felt most supported when they had clear guidance from knowledgeable providers who took them seriously and were willing to be flexible to accommodate ongoing symptoms.

##### Quality care is interprofessional and intermodal

Programs addressing Long COVID symptoms using a multidisciplinary approach (i.e., Workers’ Compensation, BreatheWell Physiotherapy) were perceived as higher quality than programs focused solely on pulmonary symptoms. Many participants also expressed preferences for mode of care delivery. Some felt in-person care was more appropriate as they felt seen and heard, received more ‘hands on’ assessment or therapy, and felt they were kept more accountable. Others preferred receiving care completely virtually to decrease risk of repeated COVID-19 exposure and to prevent symptom exacerbation that may be triggered by attending an in-person appointment. Some participants discovered the utility of virtual care after having Long COVID and therefore preferred a hybrid model.

“At first, I was fine with virtual care. I think it’s very handy to be able to speak for doctor over the telephone especially in my state where if I can’t get someone to give me a ride and help me down the stairs, I can't make it. So I think there is a definite utility to virtual care, but … I think … there's an aspect of when it's virtual there's this disconnect of you don’t see me hunched over wheezing with a hollow look in my eye.” (male participant, recruited through news article)

##### Quality care is relevant

Participants spoke about their perceptions of the relevance of peer support groups, certain types of rehabilitation, and self-management information received. Some participants liked patient-led, social media based Long COVID peer support groups (i.e., Facebook groups where patients shared positive and negative experiences of how their journeys were progressing) as they found them highly relevant and made them feel they were not alone in their journey. Others did not and felt they negatively impacted their mental well-being. Physiotherapy to improve breathing and movement was considered the most relevant rehabilitation service for Long COVID if it was provided safely [23]. Participants who underwent programs using traditional graded activity or exercise felt these caused symptom exacerbation, flare-ups, and relapses. These programs were disliked and considered less effective compared to programs focusing on the importance of rest, energy conservation, and pacing. Finally, participants felt the most relevant self-management information received was related to breathing exercises, pacing, and activity or energy management. These forms of self-management were described as most useful if supplemented with the ability to learn, practice, and reinforce these skills with the help of providers: “I think the … key … is learning how to pace … but like actually being taught what it is, why we do it and how [to] do it would have potentially made a huge difference.” (female participant, private clinic).

##### Quality care is well-timed and proportional to abilities

Some participants believed their symptoms would be less severe if they had learned about pacing or activity and energy management earlier than three months post-acute infection since they initially tried to push through their symptoms with the common perception that exercise would help to increase endurance and facilitate recovery. Over time, they came to believe this may have delayed their recovery. Some participants discussed how they disliked the high frequency and long duration of appointments as it made it difficult to balance activities and rest to pace and manage Long COVID symptoms. Due to physical limitations, attending numerous appointments (sometimes daily) was unfeasible and unmanageable. Despite challenges experienced, participants were motivated to manage the overwhelming frequency and duration of appointments since they felt desperate for any assistance.

“had I had some … background knowledge [earlier], then maybe I would have taken tinier baby steps and maybe I wouldn’t have … these huge crashes and maybe my recovery would have been quicker, but these are all maybes … I really don't know.” (female participant, Workers’ Compensation)

## Discussion

This study aimed to better understand the need for, access to, and quality of, health services by people living with Long COVID. Symptoms of Long COVID were found to be highly diverse and followed an episodic or relapsing–remitting pattern [55, 56]. Fluctuating symptoms had a profound impact on participants’ level of functioning, disability, and health. Initial access to services was dependent on guidance from providers and/or self-advocacy efforts. When navigating services, participants experienced barriers related to stigma; appointment logistics; testing and ‘normal’ results; as well as financial precarity and affordability of services. Participants discussed common likes and dislikes that led to varying perceptions of service quality. We framed these likes and dislikes as key lessons with the goal of providing the system with concrete ways to improve Long COVID care.

It has been well-established that Long COVID presents a wide range of symptoms that fluctuate over time and impact functioning, disability, and health [27, 53, 57–59]. The symptoms most frequently reported by our participants were fatigue and dyspnea. Based on data from the Living Systematic Review database [60], Aiyegbusi et al. (2021) reported the same two most frequently mentioned symptoms [57]. In a study exploring the lived experience of people with Long COVID, Humphreys et al. (2021) found participants reported ADLs as challenging, if not impossible [61]. Beyond ADLs, return-to-work is also severely affected by Long COVID. In our study, almost half of participants were unable to return-to-work due to ongoing symptoms. In a study exploring factors affecting mental health and well-being among people living with Long COVID, Burton et al. (2022) found half of their sample were unable to return-to-work [62]. Additionally, in a study documenting Long COVID patients’ lived experiences accessing and receiving healthcare, Ladds et al. (2020) found participants had limited prospects to return-to-work due to fatigue and brain fog [58]. Long COVID also impacts broader social and mental health in diverse ways [27, 57, 58, 61–64]. Aiyegbusi et al. (2021) reported participants had reduced capacity for social activities due to their ongoing symptoms and nearly half of the participants were emotionally affected by living with Long COVID [57]. Most participants in our study reported negative emotions due to adverse impacts of Long COVID on functioning and health or traumatic experiences such as not being believed when navigating the health system. Taking a trauma-informed lens when providing care to individuals with Long COVID may therefore be important in order to ensure individuals with Long COVID feel safe and empowered in their care [65].

We identified several access barriers to Long COVID services, most of which are supported by the literature. In a qualitative systematic review analyzing the experiences of people living with Long COVID and how they perceived available health services, Macpherson et al. (2022) found patients without a healthcare background found system navigation complex and challenging [27], highlighting the importance of health literacy which was discussed by our participants. This aligns with ability to perceive care from the Levesque framework [36]. Participants in our study felt stigmatized due to perceptions of being contagious, not being believed, and self-stigma or shame associated with contracting COVID-19, all of which align with ability to seek care from the Levesque framework [36]. Burton et al. (2022) discussed similar findings where participants felt as though they were avoided like the “plague” [62]. Self-stigma or shame, which led to decreased service utilization, was echoed in the literature [27, 63]. Many of our participants felt like they needed to self-advocate to access Long COVID services, which aligns with patients’ ability to engage in care from the Levesque framework [36]. Macpherson et al. (2022) and Ladds et al. (2020) noted their participants felt like they had to construct their own care pathways and advocate for themselves [27, 58]. The need to self-advocate was amplified if diagnostic assessments led to inconclusive results; a sentiment shared among people with Long COVID [62].

Several authors highlighted the Long COVID knowledge gap and how it contributed to uncertainty among providers around where to refer patients as well as potential options for symptom management [27, 58, 61, 62]. Similar to our findings, Macpherson et al. (2022) found patients seemed to understand there was limited knowledge about Long COVID but wanted providers to acknowledge that gap [27] and how it affected care coordination, which aligns with the concept of appropriateness at the system level from the Levesque framework [36]. Also connected to the concept of appropriateness [36], feeling understood, heard, believed, validated, and reassured was important to our participants and is echoed in the literature [27, 58, 62, 64, 66, 67]. Similar to our study, participants who did not feel supported in the aforementioned ways felt dismissed, disbelieved, and that they had to prove their symptoms [27, 57–59, 64, 66]. Our participants wanted more clarity on what they should be doing to help improve their Long COVID symptoms. This finding represents a gap in the approachability of services which limited patients’ ability to perceive and engage in health services [36]. Macpherson et al. (2022) and Humphreys et al. (2021) echoed this finding [27, 61]. Several studies, like ours, suggest pacing or activity and energy management are helpful approaches while graded activity or exercise interventions were harmful, often leading to symptom exacerbation [64, 66, 68, 69]. Graded activity or exercise interventions are now contraindicated in people with post-exertional symptom exacerbation such as individuals with myalgic encephalomyelitis/chronic fatigue syndrome [70] and some individuals with Long COVID.

Participants in our study had mixed views on mode of care delivery (virtual, in-person, or hybrid), which relate to the system-level access qualities of availability, accommodation, and appropriateness as well as the patient-level access qualities of ability to reach and engage with care [36]. Ladds et al. (2020) had similar findings [58]. In a study exploring the experiences of people with Long COVID and perceptions of support received, Kingstone et al. (2020) found participants preferred face-to-face assessments [64]. Aiyegbusi et al. (2021) noted participants appreciated virtual care options as they reduced in-person contact and risk for reinfection [57]. The perceived relevance of online patient-led support groups (i.e., Facebook) also seemed to be mixed [27, 62, 64, 66, 67, 71] with some finding benefits while others found them harmful, which complements our findings.

While many of our findings are supported by the literature, it is important to highlight those that are not. The first relates to financial precarity and the affordability of Long COVID services, which align with ability to pay and affordability from the Levesque framework [36]. Many of our participants were unable to work due to their Long COVID symptoms. While this facet has been established in the literature as discussed above, there has been no published literature to our knowledge discussing the impact of financial precarity (resulting from being unable to work and/or not qualifying for income supports) on being able to afford to access Long COVID services. A Canadian scoping review investigating unmet need for community-based physiotherapy found that adults with chronic conditions reported affordability as a barrier to accessing physiotherapy [72]. This left them four times more likely to access public services versus private physiotherapy services [72]. If similar patterns of financial precarity occur in the Long COVID population and the public system does not have the capacity to support these individuals, there will likely be many individuals who do not recover to the extent they would have if provided with affordable private supports. The second novel finding relates to timing of Long COVID services and/or resources. To meet the definition of Long COVID, individuals have to experience symptoms of COVID-19 for at least 3 months [4]. However, many of our participants felt they would not have experienced as many relapses if they would have received guidance on pacing and energy or activity management prior to three months post-acute infection. To our knowledge, there have been no studies investigating the impact of providing this information or related services earlier than three months post-acute infection. This represents an important gap in the literature that warrants further exploration.

### Limitations

Study limitations are recognized. First, we interviewed mainly female participants of White ethnicity, which may pose limitations to the transferability of findings. While the female gender has previously been associated with increased Long COVID risk [6–9], future research should focus on more ethnically and gender diverse populations and performing intersectional analyses to understand how access barriers may differ from those we describe. Second, we had challenges recruiting individuals living rurally limiting the comparisons that could be drawn between rurality and urbanicity in terms of access to services. Future research should ensure rural participants are justly represented as they may face unique access barriers. Third, we did not intentionally consider level of health literacy of participants during recruitment. Future research should consider level of health literacy of participants as this likely has an impact on access and utilization of health services. Fourth, selection bias may have arisen as there may have been similarities between individuals who consented to be contacted during the recruitment process. However, we had overwhelming interest and support from individuals with Long COVID as the community is keen to advance understanding. Finally, although having two individuals conduct the interviews might be perceived as impacting the consistency with which interviews were completed, we believe it was a strength because of a common interview guide and frequent meetings to review each other’s interviews. Additionally, we believe that the volume of interviews mitigated any potential negative impacts of having more than one interviewer.

### Implications for practice

We propose several recommendations to improve Long COVID services based on study findings. First, a concerted effort is needed to educate providers, especially family doctors, about Long COVID management and local Long COVID programs, referral pathways, self-management resources, and emerging evidence. Several participants noted their family doctor had no idea how to help them, which left them to explore available programs on their own. This poses a significant access barrier for people who do not have a primary care physician, are unable to advocate for themselves, or do not have the health literacy to explore programs on their own. Second, it was critical for our participants to feel as though they were not going through Long COVID alone. Support came in the form of positive therapeutic relationships with providers and peer support. Therefore, we strongly encourage providers to listen, reassure, and validate patients’ Long COVID experiences, even if they do not know how to help. We also support the development of local peer support groups to encourage personal connections and spaces of understanding. Third, we support the development of multidisciplinary clinics located in accessible urban and rural areas that tailor assessment and treatment to each Long COVID patient’s unique situation. Multidisciplinary clinics have since been created or improved and served 1897 Albertans through the public sector between June 2020 and August 2022 [73] as well as 308 Workers’ Compensation claimants between March 2021 and June 2023 [74]. Psychological support is important to help alleviate the distress associated with having a chronic illness and should be offered as part of multidisciplinary care. While providing this support would be easier in a clinical setting, ingenuity is needed to make services accessible regardless of whether someone has access to a Long COVID clinic. Psychological support should also not be mandatory or be used as a way to minimize the symptoms and experiences of individuals with Long COVID through attaching psychological diagnostic labels such as “anxiety” or “depression”. Finally, even if clinics do not see patients for initial appointments until the three-month post-acute time period (to meet the definition of Long COVID), we encourage clinics (and/or family doctors) to provide clear and simple information about the importance of pacing and energy or activity management so patients can begin practicing these techniques as soon as possible after acute COVID-19 infection, irrespective of variant [7].

## Conclusion

Our study aimed to understand the need for, access to, and quality of, health services by people living with Long COVID. With Long COVID services continuously evolving, there is an opportunity for decision makers in the health system to use our findings to better understand the experiences of those living with Long COVID and tailor services and policies appropriately. When implementing recommendations, feedback and insight should be sought from individuals with Long COVID as they are active knowledge generators and not merely passive end-users. Future research efforts should focus on evaluating the effectiveness of any service or system changes made based on our recommendations.

### Supplementary Information

**Additional file 1: Appendix A.** Participant self-identified cultural or ethic background.

**Additional file 2: Appendix B.** Full transcript quotes and shortened versions included in manuscript.

## Data Availability

The dataset generated and analyzed during the current study is not publicly available as we did not get consent from participants to share their data publicly. However, the dataset can be made available from the corresponding author on reasonable request.

## Abbreviations

COVID-19: Coronavirus Disease 2019
ID: Interpretive description
RAL: Rehabilitation Advice Line
PCR: Polymerase chain reaction
ADLs: Activities of daily living
