# Supplementary material for: “None of us are lying”: an interpretive description of the search for legitimacy and the journey to access quality health services by individuals living with Long COVID
Source: BMC Health Serv Res. 2023 Dec 12;23:1396. doi: 10.1186/s12913-023-10288-y (PMC10714615; doi:10.1186/s12913-023-10288-y)
Supplement: Supplementary file 1 — Additional file 1: Appendix A. Participant self-identified cultural or ethic background. [file 12913_2023_10288_MOESM1_ESM.docx]

Appendix A: Participant self-identified cultural or ethic background.

| **VARIABLE** | **TOTAL^b^** |
| --- | --- |
| Self-identified cultural or ethic background^a^ |  |
| Belgian | 2 |
| Black | 1 |
| Canadian | 2 |
| Chinese | 1 |
| East Indian | 3 |
| English | 2 |
| European | 1 |
| Filipino | 3 |
| French | 1 |
| Indigenous | 2 |
| Irish | 1 |
| Italian | 1 |
| Métis | 2 |
| Pakistani | 1 |
| Scottish | 1 |
| Swedish | 1 |
| Vietnamese | 1 |
| White | 39 |

^a^ Participants were asked to self-identify their cultural or ethnic background using the open-ended question “How would you describe your cultural or ethnic background?”

^b^ Total does not add to 56 as some participants identified with multiple categories
