# Supplementary material for: “None of us are lying”: an interpretive description of the search for legitimacy and the journey to access quality health services by individuals living with Long COVID
Source: BMC Health Serv Res. 2023 Dec 12;23:1396. doi: 10.1186/s12913-023-10288-y (PMC10714615; doi:10.1186/s12913-023-10288-y)
Supplement: Supplementary file 2 — Additional file 2: Appendix B. Full transcript quotes and shortened versions included in manuscript. [file 12913_2023_10288_MOESM2_ESM.docx]

Appendix B: Full transcript quotes and shortened versions included in manuscript.

| **Quote Included in Manuscript** | **Full Quote from Transcript** |
| --- | --- |
| “it affects everybody […] to different extents and everybody has different symptoms, which is really hard because it's just a myriad of symptoms” (female participant, private clinic) | “But the problem is it affects everybody differently, and to different extents and everybody has different symptoms, which is really hard because it's just a myriad of symptoms.” (female participant, private clinic) |
| “I've had to really prioritize what's the most important things to do and how I need to space that through the week. And … I have to really break down my tasks and to small steps like if I go down and start laundry, I can't make a bunch of trips going down to fill the washer because I get really short of breath and tired. If I grab laundry out of the dryer and carry it up the stairs, by the time I get upstairs I am so short of breath and exhausted, I put on my bed, and I go and I go sit my chair and I rest for about a couple hours. So … I've had to figure out ways to break up the tasks … Because … it's tiring” (female participant, Workers’ Compensation) | “I've had to really prioritize what's the most important things to do and how I need to space that through the week. And then as things, I have to really break down my tasks and to small steps like if I go down and start laundry, I can't make a bunch of trips going down to fill the washer because I get really short of breath and tired. If I grab laundry out of the dryer and carry it up the stairs, by the time I get upstairs I am so short of breath and exhausted, I put on my bed, and I go and I go sit my chair and I rest for about a couple hours. So I have to, I've had, I've had to figure out ways to break up the tasks. Whether it's put the laundry in the basket and just take it to the main level and just rest for a while and then after that carry it up the rest of the way up the stairs and rest awhile after that. Because I find even doing stuff with my arms for, like folding and lifting like you know when you do towels and stuff and everything like that, it's tiring” (female participant, Workers’ Compensation) |
| “I’m still nowhere from being able to return-to-work which is really hard to deal with. I loved my job and I really […] hoped that I could return to it, but I’m not sure […] that is going to be a reality for me […] maybe I’ll be able to return to something […] but, I need something that's going to be really flexible” (female participant, private clinic) | “I’m still nowhere from being able to return to work which is really hard to deal with. I loved my job and I really had hoped that I could return to it, but I’m not sure that that is going to be a reality for me. And so 2022 is going to be a challenging year because, if I don't return by November, I’ll lose my job, and so, when the last week and a half has been hard. It's exciting that it's a new year, and hopefully I’ll make more improvements on the beta blockers, but it's also the realization that I still have a long way to go before I’ll be able to return to a hospital and work 8:00-4:15 on paper, which is really till 5:30 or 6 without any breaks. Like I just, I’m not sure that I’m ever going to be able to do that again, which is, which is hard. And I used to teach at the university for the pediatrics course, and I really missed doing that. You know I used to do some private stuff on the side and I’ve missed doing that. But you know, maybe I’ll be able to return to something where I can still help [name of instructor] TA and still do just a little bit of private but, I need something that's going to be really flexible |
| “… it takes me two sleeping pills, three sleeping pills … to be able to go to sleep and I don't stay asleep and it's not really a deep sleep ever … I … g[e]t maybe an hour and a half of REM a night … so I just wake up exhausted … every single day.” (female participant, public clinic) | “it takes me two sleeping pills, three sleeping pills sorry to be able to go to sleep and I don't stay asleep and it's not really a deep sleep ever like if you, again, I have the fitbit so I can see what I've done during the night and everything and really I've got so much, I've got maybe an hour and a half of REM a night thinking I don't have a good sleep, so I just wake up exhausted with a horrible headache and horrible pain can move my arm, every single day.” (female participant, public clinic) |
| “it impacts … every single facet of your relationships, your ability to maintain connections outside of the home with friends, your ability to … be a father, ability to be a supportive partner and husband” (male participant, recruited through news article) | “it impacts like every single facet of your relationships, your ability to maintain connections outside of the home with friends, yeah ability to you know yeah be a father, ability to be a supportive partner and husband, yeah. Yeah.” (male participant, recruited through news article) |
| “when … your life is not normal anymore and when you have … an invisible disease … it's hard because people … can't see that you're ill … So the psychologist … has been … critical because I am not depressed, but I can see how this would push a person into a depression. I'm sad, … I'm angry, I'm frustrated … but I'm not depressed, but I have seen people [online who have] lost their jobs, …all their money, … their homes. So psych[ological] support to help people [is] critical.” (female participant, Workers’ Compensation) | “when you lose your life, your life is not normal anymore, and when you have I'm going to call this an invisible disease because if you saw me walking down the hallway I don't think you would know that I have long COVID. So to, it's hard because people assume you're better and like why you're not doing this, why you're not doing that, but they, they can't see that you're ill and you get exhausted explaining having to rationalize well I don’t feel well so I don't call my friends and I don't go out, I don't buy groceries during the week because I'm too tired after work, and you know I, why am I always going to doctor appointments? So the psychologist I think has been other than [provider] helping with the breathing and yet these other things, the psychologist has been golden, truly golden, critical because I am not depressed, but I can see how this would push a person into a depression. I'm sad, I'm sad, I'm angry, I'm frustrated that this has happened to me but I'm not depressed, but I have seen people on the COVID Facebook page who other people are trying to talk them from not committing suicide because they've lost their jobs, they've lost all their money, they've lost their homes. So psychology appointments, support to help people, critical.” (female participant, Workers’ Compensation) |
| “Even knowing about it. That's the barrier … I had no clue … I never even thought these things existed. I just thought I was … going to have to do it myself.” (female participant, recruited through news article) | “Even knowing about it. That's the barrier like until you told me about this, I had no clue I don't know that my doctor knows. I don't know if she knows. I just, I just feel like when you told me about this it's like wow like I, I never even thought these things existed. I just thought I was just going to have to do it myself.” (female participant, recruited through news article) |
| “It's … not knowing who to call. … my family doctor is fish out of water like there is no 1-800-post-COVID helpline … right? … [T]here's a lot of people out there and they're bouncing around … trying to manage on their own … just don’t know who to go to anymore” (male participant, private clinic) | “It's, it's not knowing who to call. That's like you know I, my family doctor is fish out of water like there is no 1-800 post-COVID helpline you know that kind of thing right, I mean that’s what’s really need is there's a lot of people out there and they're bouncing around. I've talked to people that have uh post-COVID and they're just bouncing around trying to manage on their own by doing things, change their diet or you know, doing things that are simple things, but you know these, these things are, are serious like you know. I know it's if you just don’t know who to go to anymore” (male participant, private clinic) |
| “I think finding the right doctors and the right people to help with … the hard part [is essential] and when was as sick as I am and was, … I just wish I didn't have to do that myself.” (female participant, Workers’ Compensation) | “I think finding the right doctors and the right people to help with the, the hard part and when was as sick as I am and was I, I just wish I didn't have to do that myself.” |
| “I was going for my follow-up … lung test … And [the tech] … looked at the requisition and … said you have COVID. … [And] in front of everybody [she said] … you have to go home … you have COVID and I was mortified … it's scary enough to have COVID. [T]hen to be treated like you have the plague after … you're not contagious anymore, … it was really very bad.” (female participant, public clinic) | “I was going for my follow up, follow up um lung test and this was probably six weeks or so after COVID maybe longer and I went to a like DynaLie, not DynaLife, MIC. And she got the requisition she looked at the requisition and she said you have COVID I said no, I didn't, I don't have COVID, I had COVID. I'm here to do my follow up, I mean she in front of everybody was blatantly loud and saying we can't do this, we can't do this, you have to go home, you have to leave you have COVID and I was mortified bawling my eyes out she says I'm going to go off and good to go and ask some people some questions, then she came back and she said again loudly we're going to do it for you, because you're here now and then I walked into the X-Ray room and as we're doing that, she says, I, I don't know why they sent you here. I said it's a follow up to see because I had like what my lungs look like ground glass. Like there was issues with my lungs and they wanted to see if it had healed or whatever what direction it was going so I said they just they wanted me to come and have a follow up she says don't talk anymore, we want you out of here as fast as possible. And everybody could hear this so it was little old people being wheeled by me in the room while she's yelling at me about having COVID and um gosh shortness of breath and um so she, like I was afraid that they were going, the old people were going to say, well, we can't stay here can’t be here and then whatever they came for, they couldn't do because of how she was you know saying I have COVID and so, anyway, it was just horrible, it was mortifying, it was embarrassed as anything. My doctor told me to write a letter to them, I did, they actually they responded to me and everything and they reprimanded her and apparently she got fired so, but it was just I mean, first of all it's scary enough to have COVID then to be treated like you have the plague after you're you know you're not contagious anymore, you know it was really very bad.” (female participant, public clinic) |
| “Some … things were virtual so that really helped me [so I didn’t] have to drag [my] butt out there because getting up in the morning is really hard. And then as you go through the week, you're just more and more tired as you go to the things” (female participant, Workers’ Compensation) | “Some of the things were virtual so that really helped me when you have to drag your butt out there because getting up in the morning is really hard. And then as you go through the week, you're just more and more tired as you go to the things” (female participant, Workers’ Compensation) |
| “I had [all] those [tests] done and, like so many others, … these tests are showing basically nothing. And it’s like, ‘oh, your heart, … looks … fine.’ But I’m here to tell you it’s not. None of us are lying. … the tests … that you're using are ineffective … It doesn't mean that we don't have this, it just means that you don't have the means to detect it.” (male participant, recruited through news article) | “I had, after that emergency incident or was is it before that? I already had that booked. I had an ECHO. Echocardiogram, bloodwork, and a lung function test. I had I had those done and, like so many others, that um, you know, on a Facebook group Long Haul Canada. And like just about everybody else, these tests are showing basically nothing. And it’s like, “oh, your heart, and you know, it looks like it's fine.” But I’m here to tell you it’s not. None of us are lying. It’s- I’m sorry, but the tests, you know, that you're using are ineffective in finding what the heck’s going on right. It doesn't mean that we don't have this, it just means that you don't have the means to detect it.” (male participant, recruited through news article) |
| “I’m … still making [Workers’ Compensation] money which is less than what I was making before to the point where … our budget, we only have like $300 … to spare and most of that is go[ing] to covering overdue and late charges on bills that we've put off because we didn't have the money to pay them … Like we're stretched extremely thin” (male participant, Workers’ Compensation) | “I'm still looking at till May when my restrictions are lifted, still making WCB money which is less than what I was making before to the point where like I mentioned me and my wife, our budget, we only have like $300 that we have, have to spare and most of that is gone to covering overdue and late charges on bills that we've put off because we didn't have the money to pay them or emergency medical bills, like my daughter's dental appointments, or Christmas or birthdays or school fees that just pop up. Like we're stretched extremely thin” (male participant, Workers’ Compensation) |
| “nobody really knows right? Everybody was still just trying … and [saying] we'll try this and we'll do that, we'll do this, so I think, for me because … I'm so scientific and … like to have black and white answers, I’m like if I do this, I want this outcome right, so I think that was hard” (female participant, Workers’ Compensation) | “Well and nobody really knows right? Everybody was still just trying to like try this and we'll try this and we'll do that, we'll do this, so I think, for me because I'm always, I'm so scientific and I'm like to have black and white answers, I’m like if I do this, I want this outcome right, so I think that was hard” (female participant, Workers’ Compensation) |
| “she sent me so much stuff by email I didn’t know if I was coming or going. Like there was pages and pages and pages of stuff and some of it was out of my realm of understanding, … I didn't read a lot of that stuff … because I found [it] … hard to wade … through” (male participant, RAL) | “she sent me so much stuff be by email I didn’t know if I was coming and going. Like there was pages and pages and pages of stuff and some of it was out of my realm of understanding, because, because there was some more medical stuff in there and like I’m not, I’m not stupid I just, I went through some of that stuff and I’m going like how, I can’t take any of this, it’s no use in reading because it’s not sticking in my head it's meaning nothing to me so, so I didn't read a lot of that stuff from them because I found that to be very boring and hard to wade my way through it” (male participant, RAL) |
| “the message that I would like to communicate to all health care providers out there is to listen to your patients and the symptoms that they're experiencing, and don't disregard them, and don't tell them that it's just in their heads. The anxiety … that lots of people are feeling isn't what caused their illness. It's their illness and people not believing them that causes more anxiety.” (female participant, private clinic) | “the message that I would like to communicate to all health care providers out there is to listen to your patients and the symptoms that they're experiencing, and don't disregard them, and don't tell them that it's just in their heads. The anxiety and depression that lots of people are feeling isn't what caused their illness. It's their illness and people not believing them that causes more anxiety.” (female participant, private clinic) |
| “At first, I was fine with virtual care. I think it’s very handy to be able to speak for doctor over the telephone especially in my state where if I can’t get someone to give me a ride and help me down the stairs, I can't make it. So I think there is a definite utility to virtual care, but … I think … there's an aspect of when it's virtual there's this disconnect of you don’t see me hunched over wheezing with a hollow look in my eye.” (male participant, recruited through news article) | “At first I was fine with virtual care. I think it’s very handy to be able to speak for doctor over the telephone especially in my state where if I can’t get someone to give me a ride and help me down the stairs, I can't make it. So I think there is a definite utility to virtual care, but it says stigma creeping and again where I'll be on the sony describing to a doctor what I've got going on and they'll say well it’s probably asthma and I'll say no it's not asthma, I have all these tests and they’ve concluded that Salbutamol is less than 2% effective on me in the tests I’ve taken and I clearly don't show signs of asthma, the specialists have told me that and they'll say, well we don't know for sure, we’ll get you tested again and it's like I wish they would just believe me you know what I mean? I think there's, there's an aspect of when it's virtual there's this disconnect of you don’t see me hunched over wheezing with a hollow look in my eye.” |
| “I think the … key … is learning how to pace … but like actually being taught what it is, why we do it and how [to] do it would have potentially made a huge difference.” (female participant, private clinic) | “I think the other key that would have been hugely beneficial is learning how to pace early on, but like actually being taught what it is, why we do it and how we do it would have potentially made a huge difference.” (female participant, private clinic) |
| “had I had some … background knowledge [earlier], then maybe I would have taken tinier baby steps and maybe I wouldn’t have … these huge crashes and maybe my recovery would have been quicker, but these are all maybes … I really don't know.” (female participant, Workers’ Compensation) | “had I had some knowledge background knowledge, then maybe I would have taken tinier baby steps and maybe I wouldn’t have you know these huge crashes, and maybe my recovery would have been quicker, but these are all maybes right, I really don't know.” (female participant, Workers’ Compensation) |
